# Mono- and bimetallic (Pt/Cu) titanium(IV) oxide photocatalysts. Physicochemical and photocatalytic data of magnetic nanocomposites’ shell

**DOI:** 10.1016/j.dib.2020.105814

**Published:** 2020-06-04

**Authors:** Zuzanna Bielan, Ewa Kowalska, Szymon Dudziak, Kunlei Wang, Bunsho Ohtani, Anna Zielińska-Jurek

**Affiliations:** 1 Department of Process Engineering and Chemical Technology, Chemical Faculty, Gdansk University of Technology, 80-233 Gdansk, Poland; 2 Institute for Catalysis (ICAT), Hokkaido University, N21, W10, 001-0021 Sapporo, Japan

**Keywords:** bimetallic nanoparticles, copper, core-shell structure, magnetic photocatalysts, platinum, surface modification, titania

## Abstract

Surface modification of titania with noble and semi-noble metals resulted in significant enhancement of photocatalytic activity. Presented data, showing the photocatalytic properties of TiO_2_-M (where M is Pt and/or Cu) photocatalysts were further used as Fe_3_O_4_@SiO_2_/TiO_2_-M magnetic nanocomposites shells in "Mono- and bimetallic (Pt/Cu) titanium(IV) oxide core-shell photocatalysts with Vis light activity and magnetic separability" [1]. Platinum and copper were photodeposited on four different titania matrices (commercial and self-obtained ones). The prepared photocatalysts were characterized by X-ray diffraction (XRD) analysis, specific surface area measurements using the Brunauer-Emmet-Teller (BET) isotherm, diffuse reflectance spectroscopy (DR-UV/Vis) analysis as well as scanning transmission electron microscopy (STEM) analysis. Photocatalytic properties were investigated in three different reactions: H_2_ generation, acetic acid oxidation to CO_2_, and phenol degradation.

Specifications table

**Table:** 

Subject	Catalysis
Specific subject area	Photocatalytic pollutants degradation
Type of data	Tables Figures
How data were acquired	X-ray diffractometer (Rigaku Intelligent X-ray diffraction system SmartLab); specific surface analyser with BET method (Micromeritics Gemini V); diffuse reflectance spectrometer (JASCO V-670) equipped with a PIN-757 integrating sphere; high-performance liquid chromatograph (Shimadzu LC-20AD); gas chromatograph (Shimadzu GC-8A); total organic carbon analyser (Shimadzu TOC-L)
Data format	Raw Analyzed
Parameters for data collection	XRD: 2θ range of 5-80°, scan speed 1°•min^−1^, scan step 0.01° DR-UV/Vis: 200-800 nm scan BET: temperature of 77 K (liquid nitrogen temperature) Photocatalytic tests parameters are presented in detail in Experimental Design, Materials, and Methods section.
Description of data collection	TiO_2_-M photocatalysts samples where obtained using photodeposition method from metal precursors in methanol: water (vol% 50:50) mixture. Irradiation was carried out for 1 hour using mercury lamp. Obtained TiO_2_-M precipitate was dried at 80°C and calcined at 400°C for 2 hours. Detailed description of conducted researches is presented in Experimental Design, Materials, and Methods section.
Data source location	Department of Process Engineering and Chemical Technology, Chemical Faculty, Gdansk University of Technology, Gdansk, Poland Institute for Catalysis (ICAT), Hokkaido University, Sapporo, Japan
Data accessibility	With the article
Related research article	Z. Bielan, E. Kowalska, S. Dudziak, K. Wang, B. Ohtani, A. Zielińska-Jurek; Mono- and bimetallic (Pt/Cu) titanium(IV) oxide core-shell photocatalysts with UV/Vis light activity and magnetic separability; Catalysis Today; In Press [1]

## Value of the data

• Physicochemical and photocatalytic characterization of mono- and bimetallic TiO_2_ matrices complement the analysis of magnetic Fe_3_O_4_@SiO_2_/TiO_2_-M nanocomposites.
• Data presents new information in the field of titania modification with noble and semi-noble metals.
• A multitude of obtained samples allows the designation of an overall trend of photocatalytic activity for different TiO_2_ matrices.

## 1 Data Description

### 1.1 XRD analysis (Fig. 1 a-b; Table 1, Table 2, Table 3)

**Fig. 1:**
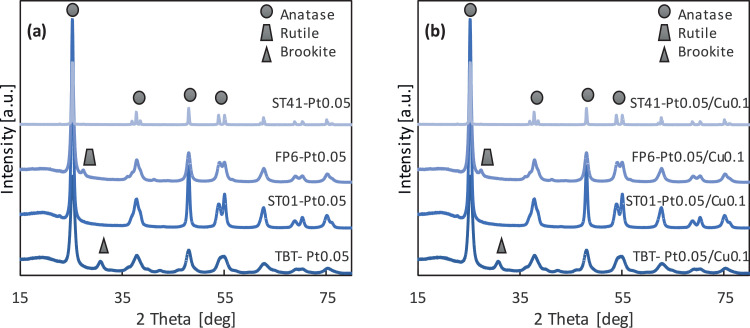
XRD patterns for monometal (a) and bimetal (b) TiO_2_-M

Exemplary XRD patterns for TiO_2_-M samples are presented in Fig. 1 a-b with detailed phase composition and crystallite sizes for all samples being listed in Table 1, Table 2, Table 3.

**Table 1:** Phase percentage and crystallite size for no-metal TiO_2_ photocatalysts

		Anatase [nm]	Anatase [%]	Rutile [nm]	Rutile [%]	Brookite [nm]	Brookite [%]
TiO_2_	TBT	8.17 ± 0.04	63 ± 6	-	-	7.69 ± 0.19	37 ± 4
	ST01	7.66 ± 0.05	100.0 ± 0.4	-	-	-	-
	FP6	11.32 ± 0.07	78.6 ± 0.3	4.87 ± 0.19	21.4 ± 0.9	-	-
	ST41	45.8 ± 0.2	100.0 ± 0.2	-	-	-	-

All obtained TiO_2_-based photocatalysts consisted of mainly anatase polymorph, and two of them were composed of only this phase (ST01 and ST41), whereas FP6 and TBT samples also contained rutile (21.4%) and brookite (37.0%), respectively. The crystallite sizes of anatase and brookite in TBT samples, based on the main peaks, reached approximately 8 nm (brookite) and 8.5 nm (anatase). In the case of commercial samples, the sizes of the crystallites vary from 5 nm to 55 nm. It was found that the crystallite size of titania increased slightly after modification with metals. For example, from 7.7 nm to 16.6 nm for ST01 modified with 0.05 mol% of Pt, which was caused by post-calcination. For FP6-M samples, anatase, as the dominant polymorphic form, ranges from 93 to 97% of the TiO_2_ crystalline phase. However, the pattern also showed peaks from rutile, mainly at 27.3 degree (110) (ICDD card No. 9004142). The size of rutile crystallites ranges from ca. 5 nm for pure FP6 to 14 nm for FP6-Cu0.1, whereas the anatase crystallites for all samples had a similar size of ca. 12 nm (11 nm for pure and 12.5 nm for FP6-Cu0.1). Compared to other TiO_2_ matrices, ST41 photocatalysts are characterized by the largest crystallites of ca. 45 nm. In the case of bimetallic TiO_2_ photocatalysts, similar crystalline properties to monometallic photocatalysts were obtained, as shown in Tables 2 and 3. It should be pointed out that the position of the peaks did not shift after titania modification with metals [2]. The presence of platinum and copper was not confirmed by XRD analysis (no peaks for platinum or copper) due to their low content (0.05-0.5 mol%) and nanometric size.

**Table 2:** Phase percentage and crystallite size for monometallic TiO_2_-M photocatalysts

		Anatase [nm]	Anatase [%]	Rutile [nm]	Rutile [%]	Brookite [nm]	Brookite [%]
TBT	Pt0.05	9.0 ± 1.3	64.50 ± 0.19	-	-	8.11 ± 0.06	35.5 ± 0.4
	Pt0.1	8.681 ± 0.015	67 ± 5	-	-	8.01 ± 0.05	33 ± 2
	Cu0.1	8.523 ± 0.013	62 ± 18	-	-	8.00 ± 0.06	38 ± 28
	Cu0.5	8.481 ± 0.012	68.2 ± 0.3	-	-	7.81 ± 0.06	31.8 ± 0.4
ST01	Pt0.05	16.58 ± 0.04	100 ± 5	-	-	-	-
	Pt0.1	16.44 ± 0.09	100 ± 1	-	-	-	-
	Cu0.1	16.01 ± 0.05	100 ± 2	-	-	-	-
	Cu0.5	15.25 ± 0.04	100 ± 8	-	-	-	-
FP6	Pt0.05	12.55 ± 0.02	96.2 ± 0.4	6.76 ± 0.12	3.8 ± 0.3	-	-
	Pt0.1	12.58 ± 0.02	92.5 ± 0.6	4.33 ± 0.07	7.5 ± 1.3	-	-
	Cu0.1	12.37 ± 0.02	97.1 ± 1.9	14.4 ± 0.3	2.9 ± 0.6	-	-
	Cu0.5	12.12 ± 0.02	96.8 ± 0.6	13.9 ± 0.4	3.2 ± 0.6	-	-
ST41	Pt0.05	54.0 ± 0.8	100.0 ± 0.2	-	-	-	-
	Pt0.1	41.42 ± 0.19	100.0 ± 0.2	-	-	-	-
	Cu0.1	45.5 ± 0.2	100.0 ± 0.2	-	-	-	-
	Cu0.5	40.2 ± 0.2	100.0 ± 0.2	-	-	-	-

**Table 3:** Phase percentage and crystallite size for bimetallic TiO_2_-M photocatalysts.

		Anatase [nm]	Anatase [%]	Rutile [nm]	Rutile [%]	Brookite [nm]	Brookite [%]
TBT	Pt0.05/Cu0.1	10.48 ± 0.05	65 ± 5	-	-	8.0 ± 0.2	35 ± 2
	Pt0.1/Cu0.1	8.81 ± 0.04	68 ± 7	-	-	8.01 ± 0.17	32 ± 4
	Pt0.05/Cu0.5	10.29 ± 0.05	68 ± 2	-	-	8.0 ± 0.2	32 ± 3
ST01	Pt0.05/Cu0.1	14.38 ± 0.06	100.0 ± 0.3	-	-	-	-
	Pt0.1/Cu0.1	14.69 ± 0.06	100.0 ± 0.3	-	-	-	-
	Pt0.05/Cu0.5	13.97 ± 0.03	100.0 ± 0.3	-	-	-	-
FP6	Pt0.05/Cu0.1	11.35 ± 0.06	94.8 ± 0.3	10.0 ± 0.4	5.20 ± 0.19	-	-
	Pt0.1/Cu0.1	11.45 ± 0.06	89.1 ± 0.3	6.5 ± 0.4	10.9 ± 0.5	-	-
	Pt0.05/Cu0.5	11.41 ± 0.06	82.2 ± 0.4	4.4 ± 0.1	17.8 ± 1.2	-	-
ST41	Pt0.05/Cu0.1	46.6 ± 0.2	100.0 ± 0.2	-	-	-	-
	Pt0.1/Cu0.1	47.0 ± 0.2	100.0 ± 0.2	-	-	-	-
	Pt0.05/Cu0.5	47.5 ± 0.2	100.0 ± 0.2	-	-	-	-

### 1.2 DR-UV/Vis spectroscopy (Fig. 2 a-b)

**Fig. 2:**
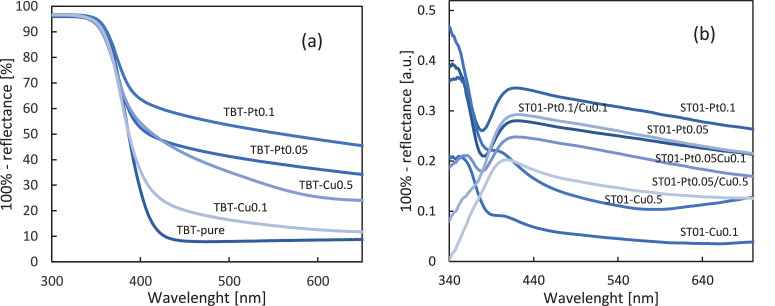
Exemplary DR-UV/Vis spectra of nanocomposites taken with BaSO_4_ (a) and pure ST01 (b) as reference.

Photoabsorption properties of no- and metal-modified TiO_2_ samples were studied by diffuse reflectance spectroscopy, and exemplary data are shown in Fig. 2 a-b.

All samples absorb UV light due to titania presence with an absorption edge at ca. 400 nm, with no difference among each polymorphic phase. The presence of noble metals resulted in the appearance of Vis absorption, as clearly shown for TBT samples in Fig. 2 a. An increase in absorption associated with the surface modification with metals is proportional to the amount of the specific type of metal used, with platinum modification resulting in a more significant increase in absorption than modification with an analogous amount of copper. Besides, for TiO_2_-Cu photocatalysts, especially when 0.5 mol% of copper was used, the spectra rises above 600 nm what is characteristic for the presence of Cu^2+^ [3]. DR-UV/Vis plots for bimetallic TiO_2_ photocatalysts are analogous to the spectra for monometallic TiO_2_.

The presence of LSPR peaks for Pt and Cu was confirmed based on DR-UV/Vis spectra measured for ST01 mono- and bimetallic photocatalysts with pure ST01 as a reference as presented in Fig. 2 b. Surface plasmon resonance of platinum is visible through an increase of the absorption in the range of about 420-440 nm [4]. Increased absorption intensity from 400 to 450 nm corresponds to an electron transfer between Cu(II) and valence band of titanium(IV) oxide or due to the presence of Cu(I). The lack of evident peak at 500-580 absorption region (typical for LSPR of Cu) indicated that zero-valent copper (photodeposited on titania surface) was easily oxidized to other forms of copper [5], which is typical for Cu-modified titania kept under ambient conditions [6].

For all obtained TiO_2_-M photocatalysts, bandgap, calculated from Kubelka-Munk transformation, was similar to the unmodified TiO_2_ (ca. 3.2 eV).

### 1.3 BET surface area analysis (Table 4, Table 5)

The specific surface area (BET) for the obtained no-, mono- and bimetallic TiO_2_ photocatalysts are presented in Tables 4 and 5. The specific surface areas of bare (10 m^2^•g^−1^ for ST41, 104 m^2^•g^−1^ for FP6, 118 m^2^•g^−1^ for TBT and 181 m^2^•g^−1^ for ST01) and metal-modified titania samples correlate well with crystallite sizes of anatase (approximately 46, 11, 8 and 7.5 nm, respectively). It was found that the metal presence caused a slight decrease in BET (Table 4) for ST01 and FP6 samples.

**Table 4:** BET surface area for no- and monometallic TiO_2_ photocalysts

TiO_2_ matrix	BET surface area [m^2^•g^−1^]				
	No-metal	Pt0.05	Pt0.1	Cu0.1	Cu0.5
TBT	118	113	112	118	100
ST01	181	113	106	113	116
FP6	104	86	81	86	88
ST41	10	10	10	10	10

**Table 5:** BET surface area measurements for bimetallic TiO_2_ photocalysts

TiO_2_ matrix	BET surface area [m^2^•g^−1^]		
	Pt0.05/Cu0.1	Pt0.1/Cu0.1	Pt0.05/Cu0.5
TBT	107	112	108
ST01	116	107	117
FP6	86	86	90
ST41	10	11	11

### 1.4 STEM analysis (Fig. 3)

**Fig. 3:**
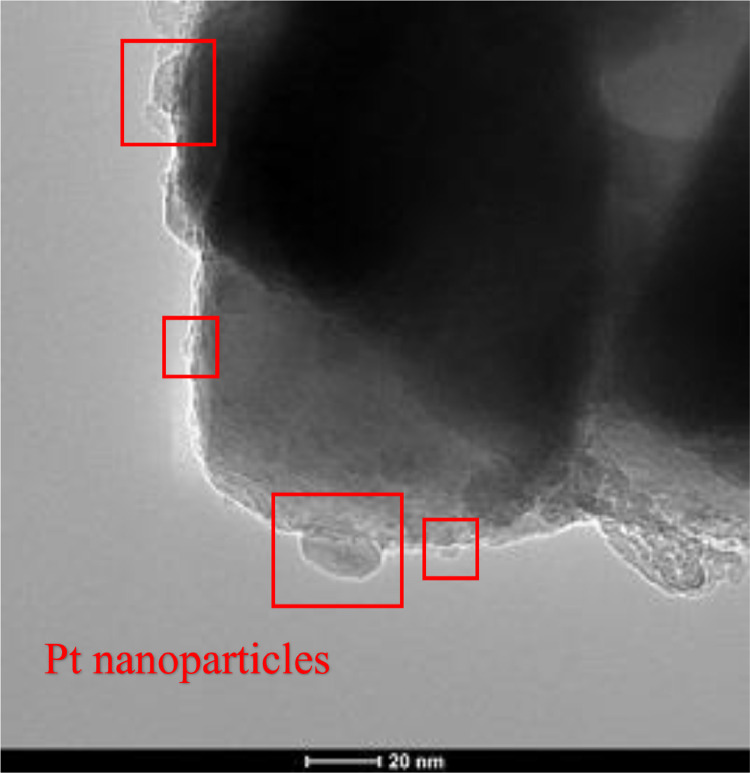
STEM images of ST01-Pt0.05 sample

For confirmation of metal presence, STEM analysis was performed. As exemplary photocatalyst, ST01-Pt0.05 was selected. The obtained images made in the dark mode are presented in Fig. 3. Platinum nanoparticles, which diameter is up to 20 nm, are marked with red squares.

### 1.5 Photocatalytic activity of mono- and bimetallic TiO_2_ (Fig. 4-8)

**Fig. 4:**
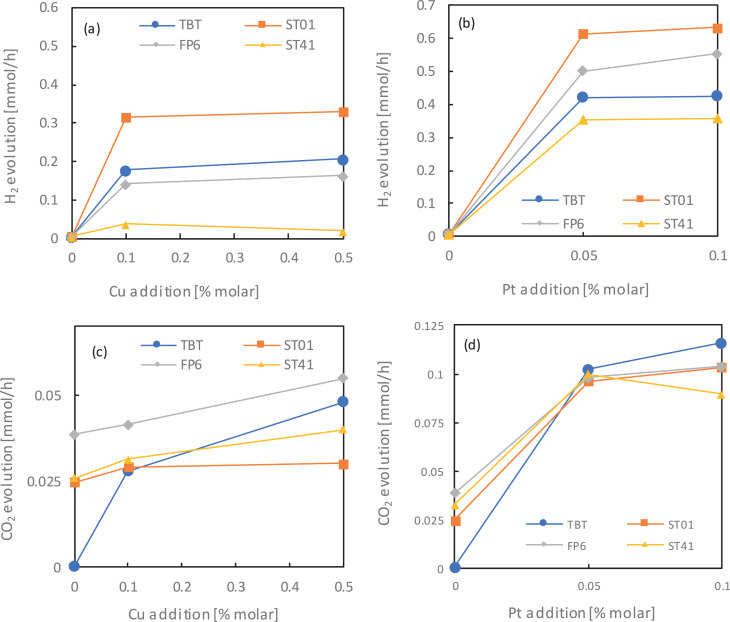
The effect of metal content on the photocatalytic activity for: (a-b) methanol dehydrogenation (H_2_ evolution), and (c-d) acetic acid decomposition (CO_2_ evolution) for different monometallic titania samples

Before evaluation of the photocatalytic activity of obtained nanocomposites [1], series of pure TiO_2_ matrices and mono- and bimetallic TiO_2_-M photocatalysts were tested as to their reference. Obtained results, presented as H_2_ and CO_2_ evolution, are shown in Fig. 4 a-d with respect on the TiO_2_ matrix as well as the amount of deposited platinum and copped.

Platinum nanoparticles significantly increase photocatalytic activity for both reaction systems, with the highest difference observed between unmodified and 0.05% Pt addition, due to the formation of Schottky barriers hindering the charge carriers’ recombination. The ST01 samples exhibited the highest activity towards hydrogen generation and the worst one for acetic acid oxidation, whereas ST41 samples behaved oppositely in both cases. It has already been reported (for comparison of 35 commercial titania photocatalysts) that for methanol dehydrogenation, a high specific surface area (ST01) for efficient adsorption of methanol is required, whereas low BET favoured acetic acid decomposition (ST41) [7]. Photocatalysts obtained from TBT and FP6 generally achieved similar results towards both H_2_ generation and CH_3_OOH oxidation. However, a little difference is still present, especially for the effect of Pt modification (Fig. 4 b). An interesting observation was also made for photocatalytic activity dependence for both platinum and copper amounts used for TiO_2_ surface modification. There was hardly any difference in H_2_ and CO_2_ generation quantity between 0.05 and 0.1% for Pt- and 0.1 and 0.5% for Cu-modified titania. Similar results were presented by Ahmed et al. [8]. The optimum platinum amount for titania modification was 0.5 wt.%, which was confirmed by methanol dehydrogenation reaction.

**Fig. 5:**
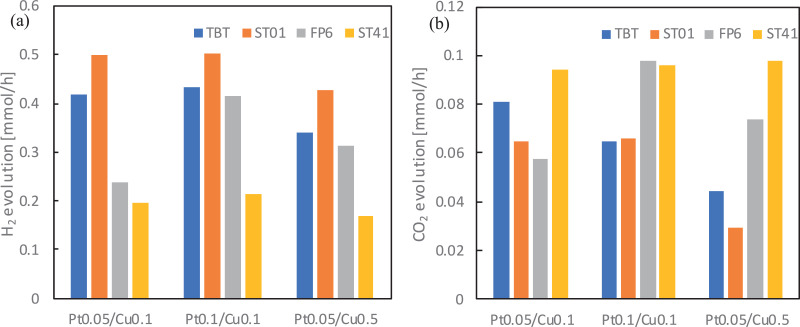
The effect of metal content on the photocatalytic activity for: (a) methanol dehydrogenation (H_2_ evolution) and (b) acetic acid decomposition (CO_2_ evolution)

The similar analysis was performed for bimetallic TiO_2_-Pt/Cu photocatalysts. Obtained results for H_2_ and CO_2_ liberation are presented in Fig. 5 a-b.

**Fig. 6:**
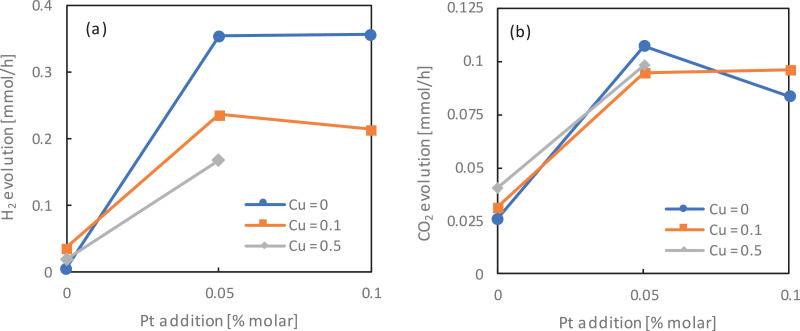
The relationship between Pt and Cu in (a) methanol dehydrogenation (H_2_ evolution) and (b) acetic acid decomposition (CO_2_ evolution) for bimetallic-modified ST41 titania samples

Analogical dependence between used titania matrix as for monometallic TiO_2_ was observed after the photodeposition of platinum and copper on the titania surface, thereby creating bimetallic structures. In reduction reaction, the ST01 matrix (containing only small anatase particles) was the most active, while in oxidation reaction – ST41 (only big anatase particles). The described dependence occurred regardless of the amount of modifying metals. For further analysis, the relationship between the platinum and copper content on ST41 matrix on photocatalytic activity in reduction and oxidation reactions is shown in Fig. 6 a-b.

As it was presented in Fig. 6 b, a combination of Pt and Cu nanoparticles on ST41 photocatalyst has hardly any influence on acetic acid oxidation. It follows that in the presented system, CH_3_OOH decomposition to carbon(IV) oxide mainly depends on the TiO_2_ matrix, to a lesser extent, from the type and concentration of metals. In reduction reaction, as it is presented in Fig. 6 a, simultaneous modification of titania surface with both Pt and Cu nanoparticles resulted in a significant decrease of H_2_ evolution, mainly because of copper introduction. The higher the mol percentage of copper used, the lower the photocatalytic activity of bimetallic TiO_2_-M.

**Fig. 7:**
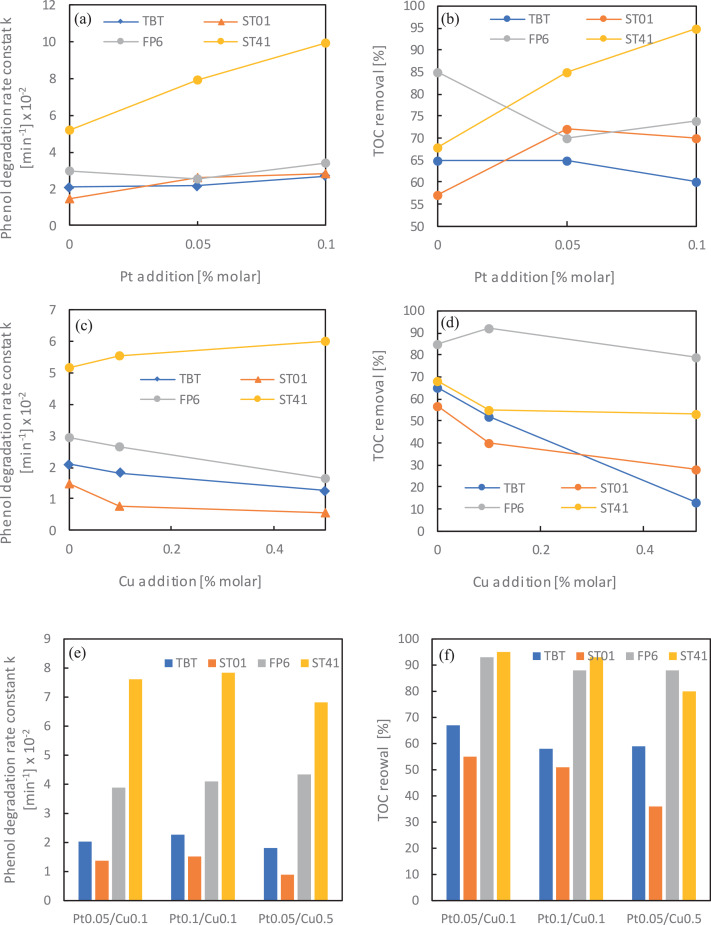
Phenol degradation, presented as a rate constant k and TOC removal for different monometallic (a-d) and bimetallic (e-f) TiO_2_-based photocatalysts

For further analysis of the photocatalytic activity of mono- and bimetallic TiO_2_ samples, phenol degradation reaction was studied. Obtained results presented as rate constant k and TOC removal are shown in Fig. 7 a-d.

In all tested systems, ST41 matrices were the most active ones, allowing for both – the highest phenol degradation as well as the highest organic carbon mineralization. The increase of the platinum content caused an increase in photocatalytic activity, while the opposite trend was observed for copper nanoparticles.

## 2 Experimental Design, Materials, and Methods

### 2.1 Materials

Commercial titania samples: ST01 (ST-01, Ishihara Sangyo, Osaka, Japan), ST41 (ST-41, Ishihara Sangyo) and FP6 (Showa Denko K.K., Tokyo, Japan) were supplied as photocatalysts matrix. Other chemicals, including titanium n-butoxide (TBT, 96.0%), chloroplatinic acid hexahydrate (99%), copper(II) sulfate (99.9%), methanol, acetic acid, acetonitrile (HPLC grade), phosphoric acid (HPLC grade, 85%) and phenol (99.5%) were purchased by Wako Pure Chemicals (Osaka, Japan). All materials were used as received without further purification.

### 2.2 Preparation of TiO_2_-M photocatalysts

Four different types of titania (commercial: ST01, ST41, and FP6, and self-prepared TBT - from titanium n-butoxide hydrolysis) were modified with platinum and/or copper nanoparticles using photodeposition method. TiO_2_ was dispersed in methanol-water solution (volume ratio 50:50) and the corresponding amount of Pt/Cu precursors’ solutions (0.05 and 0.1 mol% of Pt and 0.1 and 0.5 mol% of Cu in respect to TiO2) were added. The obtained suspension was bubbled with argon for oxygen removal. The reaction tube was sealed with a rubber septum and then irradiated for 1 h using a mercury lamp. Repeatable conditions were provided by continuous stirring (500 rpm) and temperature control using a thermostated water bath. The efficiency of photodeposition was controlled via hydrogen generation measurements taken every 15 min of irradiation. The obtained TiO2-M photocatalysts, where M corresponds to Pt, Cu, or Pt/Cu nanoparticles, were washed with deionized water, centrifugally separated, and dried at 80°C for 24 h. Finally, samples were calcinated at 400°C for 2 h.

### 2.3 Characterization of obtained photocatalysts

XRD analyses were performed using the Rigaku Intelligent X-ray diffraction system SmartLab (Tokyo, Japan) equipped with a sealed tube X-ray generator (a copper target; operated at 40 kV and 30 mA). Data were collected in the 2θ range of 5-80^o^. Scan speed and scan step were fixed at 1^o^•min^−1^ and 0.01^o^, respectively. The analysis was based on the International Centre for Diffraction Data (ICDD) database. The crystallite size of the photocatalysts in the vertical direction to the corresponding lattice plane was determined using Scherrer's equation, with Scherrer's constant equals 0.891. Quantitative analysis, including phase composition with standard deviation, was calculated using the Reference Intensity Ratio (RIR) method from the most intensive independent peak of each phase.

Nitrogen adsorption-desorption isotherms (BET method for the specific surface area) were recorded using the Micromeritics Gemini V (model 2365) (Norcross, GA, USA) instrument at 77 K (liquid nitrogen temperature).

Diffuse reflectance (DR) spectra were measured, and the data were converted to obtain absorption spectra. The bandgap energy of photocatalysts was calculated from the corresponding Kubelka-Munk function, $F{\left(R\right)}^{0.5}E_{ph}^{0.5}$ against *E_ph_*, where *E_ph_* is photon energy. The measurements were carried out on JASCO V-670 (Tokyo, Japan), equipped with a PIN-757 integrating sphere. BaSO_4_ or respective bare titania were used as references.

Noble metal nanoparticles presence was determined by scanning transmission electron microscopy (STEM) equipped with energy-dispersive X-ray spectroscopy (EDS; HITACHI, HD-2000, Tokyo, Japan).

### 2.4 Photocatalytic activity analysis

Photocatalytic activity of obtained samples was evaluated in three reaction systems: (1) phenol degradation reaction under UV-Vis irradiation, (2) decomposition of acetic acid under UV-Vis irradiation, and (3) dehydrogenation of methanol under UV-Vis irradiation. For phenol degradation reaction, a 300-W xenon lamp (LOT Oriel, Darmstadt, Germany) was used. A 0.05 g (1 g•dm^−3^) of a photocatalyst, together with a 20 mg•dm^−3^ phenol solution, was added to a 50 cm^3^ quartz photoreactor with an exposure layer thickness of 3 cm, and obtained suspension was stirred in darkness for 30 min to provide adsorption-desorption stabilization. After equilibrium was established, photocatalyst suspension was irradiated (60 mW•cm^−3^) for 60 min under continuously stirring. The constant temperature of the aqueous phase was kept at 20°C using a thermostated water bath. Every 10 min of irradiation, 1.0 cm^3^ of suspension was collected and filtered through a syringe filter (pore size: 0.2 µm) for the removal of photocatalysts particles. The concentration of phenol and formed intermediates was estimated using a reversed-phase high-performance liquid chromatography (HPLC) system, equipped with a C18 chromatography column with bound residual silane groups (Phenomenex, model 00F-4435-E0) and a UV-Vis detector with a DAD photodiodes array (model SPD-M20A, Shimadzu). The tests were carried out at 45°C and under isocratic flow conditions of 0.3 cm^3^•min^−1^ and volume composition of the mobile phase of 70% acetonitrile, 29.5% water, and 0.5% orthophosphoric acid. Qualitative and quantitative analysis was performed based on measurements of relevant substance standards and using the method of an external calibration curve. Total organic carbon (TOC) was measured using the TOC-L analyzer (Shimadzu, Kyoto, Japan).

For acetic acid decomposition, 0.05 g of the photocatalyst was suspended in 5 cm^3^ of 5 vol% aqueous acetic acid solution. The 30 cm^3^ testing tube with as prepared suspension was sealed with a rubber septum and irradiated for 60 min using 400 W mercury lamp (Hamamatsu Photonics, Hamamatsu, Japan) under continuous stirring and temperature control. Every 20 min, liberated CO_2_ in a gas phase was estimated chromatographically using a Shimadzu GC-8A Chromatograph (Shimadzu Corporation, Kyoto, Japan) equipped with thermal conductivity detector (TCD) and Porapak Q column (Agilent Technologies, Santa Clara, CA, USA).

For methanol dehydrogenation, 0.05 g of the photocatalyst was suspended in 5 cm^3^ in methanol-water solution (volume ratio 50:50). The obtained suspension was first purged with argon for oxygen removal. The testing tube was sealed with a rubber septum, and irradiated for 60 min using mercury lamp (same reaction system as that used for acetic acid decomposition). Generated hydrogen was determined every 15 min using a Shimadzu GC-8A Chromatograph with TCD detector and MS-5A column (Agilent Technologies).

## Declaration of Competing Interest

The authors declare that they have no known competing financial interests or personal relationships which have, or could be perceived to have, influenced the work reported in this article.

## Appendix Supplementary materials

Image, application 1
