# Supplementary figures and images for: Mono- and bimetallic (Pt/Cu) titanium(IV) oxide photocatalysts. Physicochemical and photocatalytic data of magnetic nanocomposites’ shell
Source: Data Brief. 2020 Jun 4;31:105814. doi: 10.1016/j.dib.2020.105814 (PMC7287231; doi:10.1016/j.dib.2020.105814)

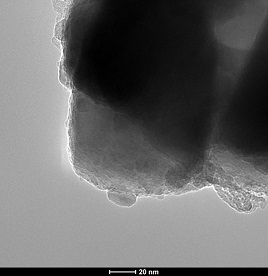

Supplement: Supplementary file 1 [file mmc1.zip › Figure 3_raw.jpg]
